# Origin of Highly Pathogenic Porcine Reproductive and Respiratory Syndrome Virus, China

**DOI:** 10.3201/eid1602.090005

**Published:** 2010-02

**Authors:** Tong-Qing An, Zhi-Jun Tian, Yan Xiao, Ran Li, Jin-Mei Peng, Tian-Chao Wei, Yi Zhang, Yan-Jun Zhou, Guang-Zhi Tong

**Affiliations:** Harbin Veterinary Research Institute, Harbin, People's Republic of China (T.-Q. An, Z.-J. Tian, Y. Xiao, R. Li, J.-M. Peng, T.-C. Wei, Y. Zhang, G.-Z. Tong); Shanghai Veterinary Research Institute, Shanghai, People's Republic of China (Y.-J. Zhou, G.-Z. Tong)

**Keywords:** Porcine reproductive and respiratory syndrome virus, highly pathogenic, origin, evolution, China, viruses, letter

**To the Editor:** A highly pathogenic porcine reproductive and respiratory syndrome virus (HP-PRRSV), which affected >2 million pigs, emerged in early 2006 in the People’s Republic of China. The disease was characterized by high fever (41°C), high illness rates (50%–100%), and high death rates (20%–100%) for pigs of all ages (*1*). A number of HP-PRRSVs have been isolated from 2006 through 2009 from infected pigs in different provinces of China and confirmed to be the causative agent of the new outbreaks (*1**,**2*). These HP-PRRSVs have a deletion of 30 amino acids in the nonstructural 2 (NSP-2). However, the evolutionary origin and path of the HP-PRRSV remain unknown.

We analyzed the full-length sequences of 67 PRRSVs: 35 HP-PRRSVs (HuN4 and LNSY-08-1 isolated in our laboratory and 33 viruses isolated in other laboratories), 28 classic PRRSVs (18 viruses isolated from China and 10 viruses representing other Asian countries and North America), and 4 commercially available attenuated live PRRSV vaccine viruses. Except for the 2 viruses we isolated (HuN4 and LNSY-08-1), the full-length sequences of the other 65 viruses were obtained from GenBank. Nucleotide and deduced amino acid sequences of these PRRSVs were aligned and compared by using previous methods (*3**,**4*).

Whole genome–based phylogenetic analysis showed that these 67 PRRSVs could be divided into 4 subgroups (Appendix Figure). Ten classic PRRSVs from China, together with the North American prototype virus VR-2332 and the vaccine virus RespPRRS/Repro modified live vaccine, were classified into subgroup 1. The first Chinese isolate, CH-1a, and its 3 derivatives (CH2002, CH2003, and CH2004) were classified into subgroup 2. All 35 HP-PRRSVs were classified into subgroup 4, and they shared high homology (>99%) in their genomic sequences. The other 4 Chinese PRRSVs, including HB-1(sh)/2002, HB-2(sh)/2002, Em2007, and SHB, belonged to subgroup 3, an intermediate subgroup between subgroups 2 and 4. Phylogenetically, HP-PRRSVs had a close relationship with subgroups 2 and 3.

Four conserved deletions were shown among all HP-PRRSVs, including an adenosine deletion at position 122 in the 5′-untranslated region, a guanosine deletion at position 15,278 in the 3′-untranslated region, and 2 discontinuous deletions in the NSP-2, including a single amino acid deletion at position 482 (L^482^) and a second deletion of 29 amino acids between positions 533 and 561 (S^533^–A^561^). The presence of these 4 deletions among subgroup 4 viruses is a unique phenomenon, which may be used as a distinctive molecular marker for HP-PRRSVs.

The occurrence of these 4 deletions might be explained as a stepwise accumulation from subgroup 2 to subgroup 4. None of the 4 deletions were found in subgroup 2. Among viruses in subgroup 3, one, 2, or 3 of the 4 deletions occurred. For example, a single deletion was present at 122 nt in Em2007, double deletions at 122 nt and 15,278 nt in HB-1(sh)/2002 and SHB, and triple deletions at 122 nt, 15,278 nt, and 482 aa in GD3-2005 (this sequence was not submitted to GenBank until now). In 2008, Ma et al. compared GD3-2005 with several PRRSVs and reported the homology within them, pointing out that the 2 deletions in NSP-2 were identical to the HP-PRRSV (*5*). After careful analysis, we found the GD3-2005 more interesting than what was reported in Ma et al.; it belongs to an intermediate group, and shares the characters of gradual evolution. Eventually, all 4 deletions occurred in subgroup 4. This obvious pattern suggests that these 4 conserved deletions might have evolved step by step.

The primary neutralizing epitope (PNE), which is located on glycoprotein 5 and composed of the residues S^37^H(F/L)QLIYN with F/L^39^ as the binding site for the neutralizing antibody (*6**,**7*), also displayed similar changes at the 39 position among the 4 subgroups. The PNE residues in subgroups 1 (SHL^39^QLIYN) and 2 (SHF^39^QLIYN) were considerably conservative. Subgroup 3 contained either F^39^ or I^39^ (F^39^ in Em2007 and HB-2(sh)/2002, and I^39^ in both HB-1(sh)/2002 and SHB); subgroup 4 contained I^39^ only. The existence of either F^39^ or I^39^ in subgroup 3 PNE indicates its intermediate position between subgroups 2 and 4 in the evolution of HP-PRRSVs.

Pairwise comparison of subgroups 2, 3, and 4 did not find recombination or large fragment replacement, which suggests that all HP-PRRSVs originated from the same ancestor by gradual evolution. Notably, the recently isolated intermediate PRRSVs mentioned above (SHB, Em2007, and GD3-2005) were isolated in the region of South China where the outbreak of HP-PRRS initially occurred. Furthermore, the epidemiologic data show that the outbreak of HP-PRRSV emerged from 1 particular place and then spread widely. This evidence indicates that all HP-PRRSVs isolated in China likely originated from the same source.

In summary, our findings suggest that the newly emerged HP-PRRSVs originated from the Chinese CH-1a-like PRRSV. Further study is needed to determine what contributes to the increased pathogenicity of HP-PRRSV. Although the 4 deletions are conserved in all HP-PRRVs, the increased pathogenicity of HP-PRRSV may not merely be caused by the deletions; pathogenicity is affected by multigenetic factors.

## Supplementary Material

Appendix Figure Phylogenetic relationships of 67 porcine reproductive and respiratory syndrome viruses (PRRSVs) based on their whole-genome sequences. The unrooted phylogenetic tree was generated by the neighbor-joining method using Molecular Evolutionary Genetics Analysis 4 (*5*). Bootstrap values were calculated on 1,000 replicates. The 53 isolates from China were classified into 4 subgroups (circled). Four commercially available attenuated live vaccine viruses are marked with asterisks. MLV, modified live vaccine; NVSL, National Veterinary Services Laboratories; CH, China; SP, Singapore; HN, Henan; BJ, Beijing; HB, Hebei; WUH, Wuhan; JX, Jiangxi; GD, Guangdong; LN, Liaoning; NM, Neimenggu; JS, Jiangsu; SH, Shanghai; TJ, Tianjin; SX, Shanxi; HUB, Hubei; YN, Yunnan; NX, Ningxia, GS, Gansu.
