# Supplementary figures and images for: Origin of Highly Pathogenic Porcine Reproductive and Respiratory Syndrome Virus, China
Source: Emerg Infect Dis. 2010 Feb;16(2):365–7. doi: 10.3201/eid1602.090005 (PMC2957991; doi:10.3201/eid1602.090005)

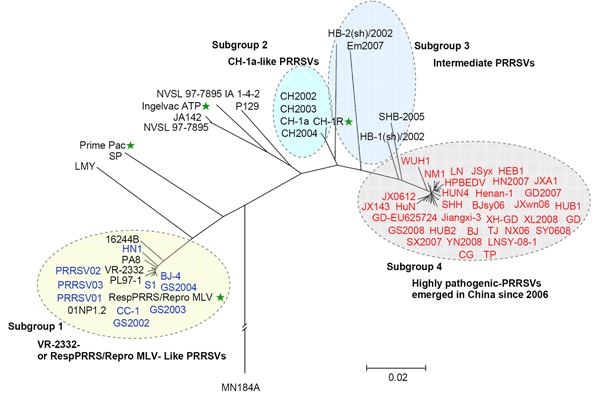

Supplement: Appendix Figure — Phylogenetic relationships of 67 porcine reproductive and respiratory syndrome viruses (PRRSVs) based on their whole-genome sequences. The unrooted phylogenetic tree was generated by the neighbor-joining method using Molecular Evolutionary Genetics Analysis 4 (5). Bootstrap values were calculated on 1,000 replicates. The 53 isolates from China were classified into 4 subgroups (circled). Four commercially available attenuated live vaccine viruses are marked with asterisks. MLV, modified live vaccine; NVSL, National Veterinary Services Laboratories; CH, China; SP, Singapore; HN, Henan; BJ, Beijing; HB, Hebei; WUH, Wuhan; JX, Jiangxi; GD, Guangdong; LN, Liaoning; NM, Neimenggu; JS, Jiangsu; SH, Shanghai; TJ, Tianjin; SX, Shanxi; HUB, Hubei; YN, Yunnan; NX, Ningxia, GS, Gansu. [file 09-0005_appF-s1.gif]
